# Repellent Screening of Selected Plant Essential Oils Against Dengue Fever Mosquitoes Using Behavior Bioassays

**DOI:** 10.1007/s13744-023-01039-z

**Published:** 2023-03-16

**Authors:** Wan Deng, Mi Li, Sisi Liu, James G. Logan, Jianchu Mo

**Affiliations:** 1 State Key Lab of Utilization of Woody Oil Resource, Research Institute of Forest and Grass Protection, Hunan Academy of Forestry, Changsha, China; 2 Ministry of Agriculture Key Lab of Molecular Biology of Crop Pathogens and Insects, Institute of Insect Sciences, Zhejiang Univ, Hangzhou, China; 3 Dept of Disease Control, London School of Hygiene and Tropical Medicine, London, UK; 4 Arctech Innovation Ltd, the Cube, Dagenham, UK

**Keywords:** Mosquito repellent, *Ae. aegypti*, *Ae. albopictus*, Aromatic plants, Natural products

## Abstract

Among the efforts to reduce mosquito-transmitted diseases, such as malaria and dengue fever, essential oils (EOs) have become increasingly popular as natural replacements for the repellant DEET. In this study, seven commercially available plant EOs against *Aedes* species mosquitoes were evaluated for their complete protection time (CPT, min) in vivo using human-hand in cage tests (GB2009/China and WHO2009). Among the EOs with the highest efficacy in repelling mosquitoes, *Aedes albopictus* (Skuse) were clove bud oil and patchouli oil. Both were further assessed according to the in vivo method recommended by the WHO, to determine their minimum effective dose and CPT. A comparison of the ED_50_ values (dose yielding a 50% repellent response) of these two EOs against *Aedes aegypti*(L.) showed that the ED_50_ (2.496 µg/cm^2^) of patchouli oil was 1248 times higher than that of clove bud oil (0.002 µg/cm^2^), thus demonstrating them greater efficacy of the latter in repelling *Ae. aegypti* mosquitoes. For the 2 EOs, eugenol was the major component with higher than 80% in relative amount of the clove bud oil. The patchouli oil had more than 30% of character chemical patchouli alcohol along with α-bulnesene (10.962%), α-guaiene (9.227%), and seychellene (7.566%). Clove bud oil was found to confer longer complete protection than patchouli oil against a common species of mosquito. These results suggest use of EOs as safe, highly potent repellents for use in daily life and against mosquito-transmitted diseases, such as malaria and dengue fever.

## Introduction

Mosquitoes are not only a nuisance in daily life, they transmit pathogens that cause several diseases, such as dengue fever and malaria, in many areas of the world, Thus far, the effective control of mosquitoes remains elusive (de Oliveira et al. 2020). As vaccines effective against mosquito-borne diseases are not yet available, with the exceptions of those against Japanese encephalitis and yellow fever (CDC 2022). Therefore, the prevention of mosquito bites remains the most common strategy to control or minimize the incidence of mosquito-transmitted diseases (da Silva and Ricci-Junior 2020; Jones et al. 2021).

Repellents are commonly used for personal protection against mosquito bites, due to their effectiveness, availability, and ease of use (Zhu et al. 2018). In general, repellents can be divided into synthetic preparations and natural plant-based products. Among the former is DEET, which has been used since the 1950s and shows good efficacy at relatively low doses. However, concerns regarding its side effects in humans, and environmental toxicity (Oliferenko et al. 2013; Slaninova et al. 2014), the insensitivity/acquired resistance of some vectors (Stanczyk et al. 2010, 2013) have greatly influenced customer acceptance; and greater interest in natural, eco-friendly plant-based repellents have been studies in the world. The latter include para-menthane-3,8-diol (PMD), a natural and safe topical repellent alternative to DEET (Grison et al. 2020).

In addition, strong repellent effects against mosquitoes have been demonstrated for a large number of essential oils (EOs) extracted from different plant families, including Poaceae, Myrtaceae, Lamiaceae, Lauraceae, Asteraceae, and Rutaceae (Lee 2018; Gharsan 2019; Osanloo et al. 2019; Al-Sarar et al. 2020; Durofil et al. 2022). The most frequently studied repellent EOs are those obtained from plant species belonging to the genera *Cymbopogon* Spreng., *Ocimum* L., and *Eucalyptus* L. Hér. spp. In many of these, the active chemical repellent has been identified (da Silva and Ricci-Junior 2020). In this study, after a literature review of 144 EOs reported to have mosquito repellent activity (unpublished), seven EOs from different plant families and approved for human testing by the ethics committee of our institution were assessed for their ability to repel *Aedes* mosquitoes in in vivo bioassays.

## Materials and methods

### Preparation of EOs

Based on a review of traditional Chinese medicine products as mosquito repellents after safety evaluation by the Research Ethics Committees, seven EOs were chosen for further testing as potential mosquito repellents (Table 1). These EOs were selected because they were previously reported to have repellent properties against mosquitoes or their chemical structures were similar to those of other components with repellent properties (Chellappandian et al. 2018; Al-Sarar et al. 2020; de Oliveira et al. 2020; Esmaili et al. 2021).

**Table 1:** The seven essential oils (EOs) selected for testing as mosquito repellents in in vivo (GB2009, WHO2009) bioassays

No	English name	Latin name	Manufacturer	Bioassay
1	Fennel	*Foeniculum vulgare* Mill	Sigma	GB2009
2	Cinnamon	*Cinnamomum zeylanicum* Blume	Sigma	GB2009
3	Ginger	*Rhizoma zingiberis* Recens	Sigma	GB2009
4	Peppermint	*Mentha haplocalyx* Briq	Sigma	GB2009
5	Black pepper	*Piper nigrum* L	Sigma	GB2009
6	Patchouli	*Pogostemon cablin* Benth	Sigma	GB2009, WHO2009
7	Clove bud	*Syzygium aromaticum* (L.) Merr.et Perry	Sigma	GB2009, WHO2009

### Test insects

Disease-free *Ae. albopictus* and *Ae. aegypti* female mosquitoes were obtained from cultures maintained at the Institute of Insect Sciences, College of Agriculture & Biotechnology, Zhejiang University (Hangzhou, China), and the London School of Hygiene and Tropical Medicine (London, UK). Laboratory cultures of the mosquitoes were maintained in a controlled environment at 27 ± 2°C, 55–60% relative humidity (RH), and a light:dark photoperiod of 12 h:12 h. Adult mosquitoes were placed in plastic rearing cages and provided with a 10% sucrose solution on cotton wool. Females were blood-fed weekly on horse blood using a Hemotek® membrane feeding system (Discovery Workshops, UK) or a homemade system. For the bioassays of repellent activity, 5- to 12-day-old female mosquitoes not previously blood-fed were collected and placed in a fresh cage, without sucrose or water, in the bioassay room for 2 h, which allowed the insects to become acclimated to their environment.

### Bioassays

#### *In vivo *tests

Two different bioassays were used to assess mosquito repellency. In the first, the seven EOs were tested according to a standard method used in China (GB2009). In the second, the two most effective EOs were further evaluated according to the WHO’s human-arm in cage test (WHO2009). GB2009 is the first method developed for the screening of potential mosquito repellents against *Ae. albopictus* which is the major species of mosquito in China. The WHO guideline 2009 was used to assess the two most effective EOs to provide a reference for researchers in comparisons with other EOs tested as mosquito repellents against *Ae. aegypti*.

#### GB2009

In this test, a 16-cm^2^ area of skin on a human hand is treated with the test or control compound and placed in a mosquito-containing test arena (described below). The limited skin-exposure area significantly reduces the risk of side effects from the bites.

#### Test arena

Three hundred female adult (4–5-days-old) mosquitoes (*Ae. albopictus*) were used in the trial. The insects were given access to sugar water prior to their use in the test but did not receive a blood meal. The experiments were conducted in a 40 cm * 30 cm * 30 cm cage maintained at 25–27°C and a RH of 60–70%.

#### Test procedure

The mosquito repellence activity of the EOs was assayed as follows. The tester’s hands were cleaned with distilled water and his/her forearms protected with a thick fabric sleeve. The tester then donned latex surgical gloves with a 4 × 4 cm area cut out to expose the skin on the dorsal side. One hand served as the control and the other as the test hand. The tester’s control hand was rubbed with 37.5 μL of ethanol (70%) and then placed for 3 min in a cage containing 300 mosquitoes. The number of probing mosquitoes was recorded. If the recorded number was > 30, the test was continued; otherwise a new population of mosquitoes was placed in the cage. The test hand was then treated with 37.5 μL of EO in ethanol (see Table 2 for the dose) and exposed to mosquitoes in the same test cage. Dissolving the EOs in alcohol improved their skin permeability (Lupi et al. 2013). Each EO concentration was tested for up to 3 min during a 15-min period, until either two bites occurred in a single exposure or one bite occurred in each of two consecutive exposures. The time between repellent application and the first two bites in a single exposure or successive exposures was defined as the complete protection time (CPT). Each concentration was tested four times. The longest time for a test did not exceed 7 h.

**Table 2:** The tested EOs and their test concentrations

No	EO	Concentration (%)						
		0.20	0.25	2.50	10	20	25	30
1	Fennel oil	★						
2	Cinnamon	★	★					
3	Peppermint	★	★	★				
4	Ginger oil	★	★	★	★			
5	Black pepper	★	★	★	★	★		
6	Patchouli oil	★	★	★	★	★	★	
7	Clove bud oil	★	★	★	★	★	★	★

Each sample was diluted with 70% ethanol to obtain the approved concentration range approved for testing

The efficacy of the EOs as repellents was assessed according to:

1. The percentage protective efficacy (PE%), calculated as PE% = [(number of probing mosquitos in the untreated vs. the treated hand)/number of probing mosquitoes of the untreated hand] × 100 (Fradin and Day 2002)
2. The CPT, calculated as the time elapsed between repellent application and the first confirmed mosquito bite. An effective mosquito bite of the treated skin of the volunteer was defined as the persistence of the mosquito on the skin for 10 s.

#### Human arm in cage test (WHO2009)

##### Test arena

One hour before the start of the test, 50 *Ae. aegypti* female mosquitoes (5–12-days-old) were placed in a 30 * 30 * 30 cm cage inside the testing room. The insects were fed with sugar water prior to the test and did not receive a blood meal. The cage was held at 25°C ± 2°C and 80% RH.

##### Test procedure

The EO or the ethanol control is applied to the forearm skin (between the wrist and elbow) of three volunteers. The exposed area (~ 600 cm^2^) is determined using the WHO formula for testing skin repellents:

$$\mathrm{Area}={~}^{1}\!\left/ \!{~}_{2}\right.\left({C}_{W}+{C}_{E}\right) \times {D}_{EW}$$

where *C*_*W*_ is the circumference of the wrist, *C*_*E*_ is the circumference of the elbow, and *D*_*EW*_ is the distance between the wrist and elbow in centimeters (cm). The remaining skin area is covered by a rubber sleeve. The dose of the tested repellent is expressed as mg/cm^2^ or mL/cm^2^ and is measured using a micropipette or balance before being applied to the exposed forearm using a gloved finger. The volunteer is instructed to avoid contact with lotions, perfumes, oils, or perfumed soaps on the day of the assay.

### Repellent activity in the WHO2009 test

#### Minimum repellent dose

The two tested EOs, patchouli oil and clove bud oil, were applied at the doses (µg/cm^2^) shown in Table 3. The maximum amount of clove bud oil was 720 mg (1200 µg/cm^2^) and that of patchouli oil 56 mg (93 µg/cm^2^). The control consisted of ethanol alone. The test was conducted during the day, from 07:00 to 17:00. Each concentration was tested six times. The tester first inserted the control arm into the cages for 30 s, and then the arm treated with the first dose of EO for 30 s. The number of landing and probing mosquitoes was recorded. Successive doses of the EOs were tested until the mosquitoes stopped biting or the maximum safe dose had been reached, or the dose applied became uncomfortably high. As a final control, the untreated arm was again placed in the cage for 30 s and the number of landing and probing mosquitoes again recorded. Landing was defined as the mosquito arriving on the volunteer’s treated skin and remaining for 10 s. Biting was defined as the mosquito probing into the skin for > 10 s. If the mosquito did not land on or bite the volunteer’s treated skin, the respective dose of EO was considered to have provided 99.9% protection.

**Table 3:** Doses of clove bud oil and patchouli oil applied to human forearm skin (exposed area: ~ 600 cm^2^)

Applied dose (µg/cm^2^)		Cumulative dose (µg/cm^2^)	
Clove bud oil	Patchouli oil	Clove bud oil	Patchouli oil
0	0	0	0
0.1	9.3	0.1	9.3
0.2	9.3	0.3	18.6
0.7	9.3	1	27.9
2	9.3	3	37.2
7	9.3	10	46.5
22	9.3	32	55.8
68	9.3	100	65.1

The percentage repellency (*R*) was calculated as follows:

$$\mathrm{R\%}=\left[\left(\mathrm{C}-\mathrm{T}\right)/\mathrm{C}\right]\times 100$$

where *C* is the mean number of mosquitoes of two control tests, i.e., *C* = (*C*_Before_ + *C*_After_)/2, and *T* is the number of mosquitoes in the treated group.

#### Complete protection time

The time between repellent application and the first two mosquito bites during the same observation period or one bite in each of two consecutive intervals was defined as the CPT. Longevity was determined by estimating the CPT of a predetermined dose applied to the skin, either the dose conferring a 100% reduction at the ED_99_ (the effective dose of a repellent required to reduce biting by 99%) or the maximum safe dose as determined in the WHO’s standard application volume of 1.67 µL/cm^2^ (1.002 mL/600 cm^2^).

First, a control was conducted in which the volunteer inserted his/her bare left arm into a cage containing 50 mosquitoes and the number of mosquitoes probing the arm after 30 s was recorded. Immediately thereafter, the volunteer inserted the treated arm into the same cage for 30 s. The number of mosquitoes probing the arm was determined as described for the control.

The test was repeated at 30-min intervals for the first 3 h, followed by hourly observations until treatment failure or until 8 h had elapsed since EO application. The CPT was defined as the occurrence of one probing event by a mosquito in a 30-s test followed by a confirmed bite within 30 min. The test was repeated with six volunteers.

#### GC–MS analysis

The GC–MS analysis was carried out with an Agilent 5975 GC-MSD system. An Innowax fused silica capillary column (DB-5, 60 m * 0.25 mm* 0.25 μm film thickness) was used with helium as the carrier gas (1.5 mL/min). The GC oven temperature was held at 50°C for 2 min after injection and then ramped to 250°C at a rate of 10°C/min, and held at 250°C for 5 min. The split ratio was set at 100:1. The injector temperature was set at 250°C. Mass spectra were recorded using 70 eV electrons in election ionization (EI) mode. The mass analyzer was scanned from m/z 35–500 amu. MS was identified on the basis of computer matching of the mass spectra with those of the Wiley and MassFinder libraries and comparison with literature data.

## Analysis

In order to find the statistical difference between the repellent effects of different essential oils, the data was analyzed by one-way ANOVA with post hoc Bonferroni test. The statistical tests were performed on SPSS software (IBM, USA).

### Dose–response (µg/cm^2^)

The minimal effective dosage repelling 50% and 95% or 99% of mosquitoes was expressed as the ED_50_, ED_95_, and ED_99_ (repellent effective dose). The data were analyzed using Probit-plane regressions, from which the ED_50_ and ED_99_ and their 95% confidence intervals (95% CI) were estimated.

### Median CPT (min)

The median CPT and 95% CI were estimated using the Kaplan–Meier survival function. The protocol for EO application was based on the CPT.

## Results

In this study the mosquito repellent efficacy of seven EOs was investigated in two in vivo tests, consisting of the GB2009 and the WHO2009 human-arm in cage test. Based on the efficacy and dose information obtained in a screening, the minimum repellent dose and the CPT of seven EOs and two EOs in a GB2009 test and a WHO2009 test, respectively, were determined using two species of *Aedes* mosquitoes.

### Repellent activity

#### CPT test results for seven EOs using the GB2009 method

**Fig. 1:**
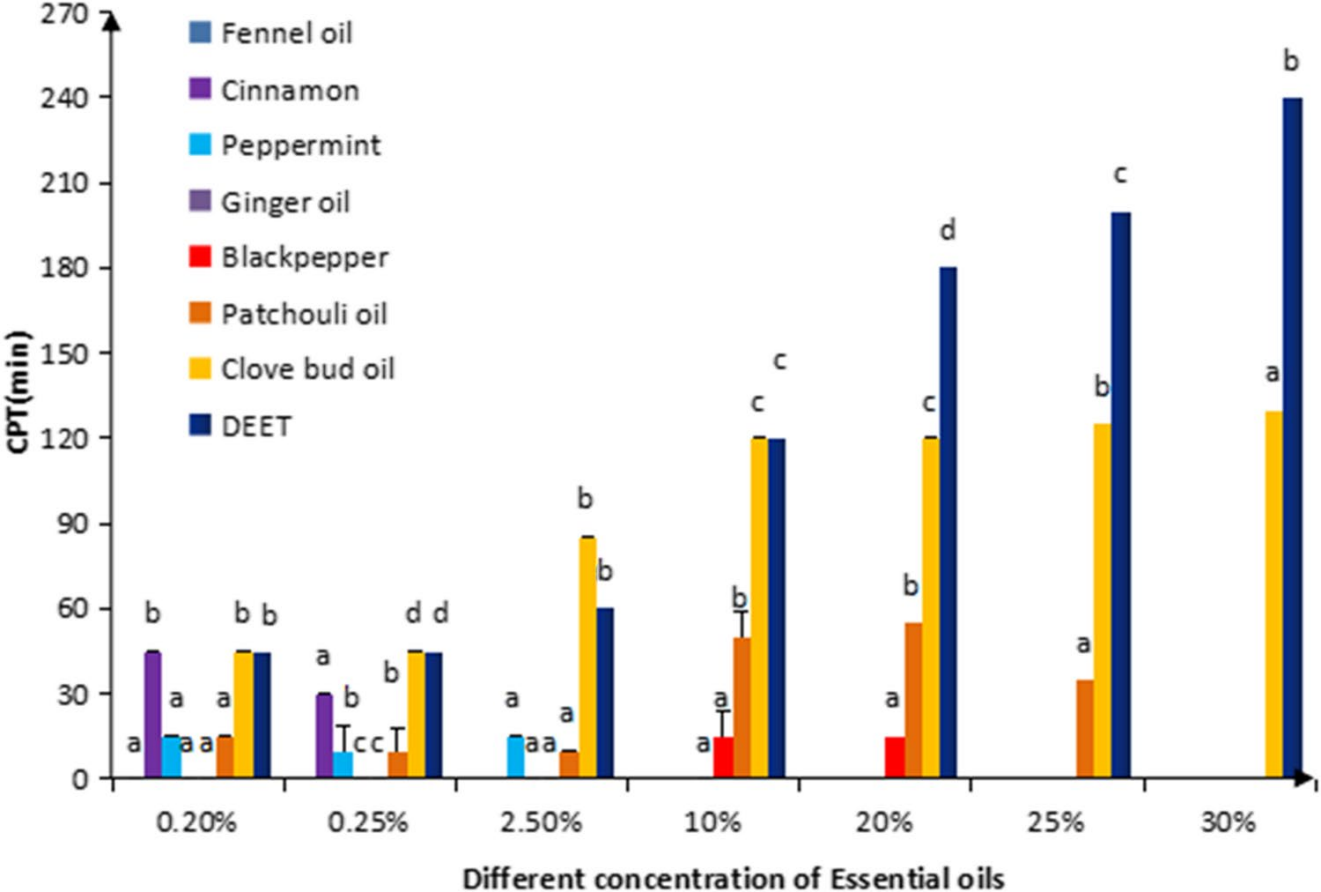
Concentration-dependent mosquito repellent persistence activity (complete persistente time - CPT) of different test EOs against female *Ae. albopictus* corresponding to their maximum safe dose as advised by the Ethics Review Committee. Different letters on bars show significant difference (*p* < 0.05) when the comparison of mosquito repellency persistence was made between different test substances after a specific concentration interval independently

Based on the literature as well as expert advice from the Ethics Review Committee, seven EOs were selected for in vivo testing using the GB2009 test against *Ae. albopictus*. Each one was tested at its specific maximum safe dose, such that the CPT was determined using the EOs at different concentrations. The results are shown in Fig. 1.

From Fig. 1, when the concentration of EOs at 0.20%, only 3 EOs of cinnamon oil, clove bud oil, and DEET got 45 min CPT and which was significantly different with other 4 EOs (*p* = 0.000). When the concentration was higher than 10%, the whole EOs had a very obvious significantly differences between each other. The best protection was obtained with clove bud oil, even at its lowest concentration. Increases in the concentration provided longer protection. At 20% of the dose of the other EOs, clove bud oil was effective for 120 min, as shown in a previous study (Trongtokit et al. 2005). But the clove bud oil still get a significantly lower CPT compared with DEET at the same concentration. The second most effective EO was 20% patchouli oil, which provided 55 min of protection. Three other EOs conferred ≤ 15 min of protection. Cinnamon oil at the lowest concentrations of 0.2% and 0.25% provided 45 min and 30 min of protection, respectively, which was comparable to the high level of protection conferred by clove bud oil at the same doses. Peppermint and black pepper oils only provided ~ 20 min of protection, even at 20%. Fennel oil and ginger oil did not repel mosquitoes at concentrations < 10%.

#### Test results using the WHO’s human-arm in cage test

**Fig. 2:**
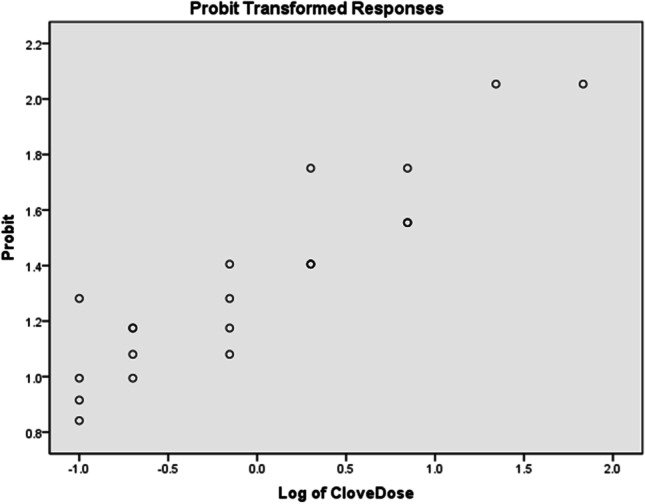
The dose–response curve of clove bud oil

**Fig. 3:**
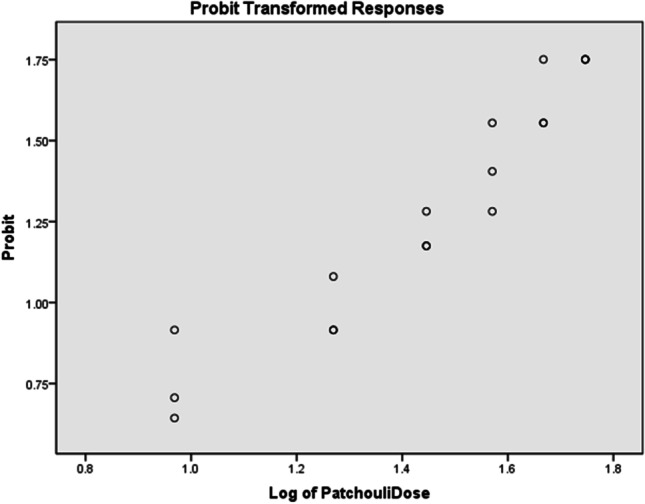
The dose–response curve of patchouli oil

To confirm the efficacies of clove bud oil and patchouli oil demonstrated in the GB2009 test, both EOs were tested against *Ae. aeygpti*, in dose–response and CPT tests performed according to the WHO’s human-arm in cage test. And the dose–response curve is shown in Figs. 2 and 3.

#### Effective dose of clove bud and patchouli oils as mosquito repellents

The effective doses of clove bud oil and patchouli oil conferring 50%, 95%, and 99% protection against *Ae. aeygpti* were determined (Kang et al. 2009). The results are shown in Table 4. Clove bud oil provided very good repellency against mosquitoes at concentrations lower than those of patchouli oil. The ED_50_ (2.496 µg/cm^2^) of patchouli oil was 1248 times higher than that of clove bud oil (0.002 µg/cm^2^). These results suggested that a low dose of clove bud oil was effective as a mosquito repellent.

**Table 4:** Repellent activity of clove bud and patchouli oils against *Ae. aegypti*

Essential oil	Safe dose (µg/cm^2^)	Repellent effective dose [95% confidence interval] (µg/cm^2^)			Median CPT [95%, confidence interval] (min)
		ED_50_	ED_95_	ED_99_	
Clove bud	1200	0.002 [0.000, 0.005]	3.833 [2.446, 6.815]	**94.292** [39.561, 335.327]	60 [53.642, 65.195]
Patchouli	**93**	2.496 [0.504, 5.068]	71.269 [49.340, 150.775]	284.679 [139.136, 1456.710]	36 [34.868, 37.132]

ED_50_, ED_95_, and ED_99_: repellent effective dose (µg/cm^2^ of skin) resulting in 50%, 95%, and 99% repulsion against *Ae. aegypti*; *CPT*, complete protection time. Doses in bold are those used in the CPT test

#### Comparable repellency of EOs

The maximum safe dose of clove bud oil according to its safety data is 1200 µg/cm^2^ of skin, which is significantly higher than the ED_99_ determined in this study (94.292 µg/cm^2^). For patchouli oil, its ED_99_ (285.679 µg/cm^2^ of skin) was significantly higher than the maximum safe dose (93.000 µg/cm^2^ of skin). Thus, in CPT tests to determine the duration of protection against mosquito bites, clove bud oil was assessed at its ED_99_ whereas patchouli was assessed at the dose conferring 96% protection (ED_96_ = 88.418 [95%CI: 58.294, 213.206]).

The results showed that the median CPT for patchouli oil (36 min [95%CI: 34.868, 37.132]) was significantly lower than that of clove bud oil (60 min [95%CI: 53.642, 65.195]), indicating longer complete protection against mosquitoes by the latter. However, while the concentrations of the two EOs applied on six volunteers were nearly the same, there was a large difference in repellent efficacy, as evidenced by the 95%CI values. Thus, the CPT for clove bud oil was the same (60 min) in all six volunteers, whereas with patchouli oil the CPT in the six volunteers ranged from 29 to 115 min (data not shown).

### GC–MS

The essential oils of clove bud oil and patchouli oil had been analyzed by using GC–MS (Table 5). And the compounds with similarity index over 90 are listed in Table 2. The major chemical was eugenol with 82.093% in the clove bud oil; the next was β-caryophyllene and acetyleugenol. The characteristic compound of patchouli oil was patchouli alcohol, accounting for 34.717% and the rest was α-bulnesene (10.962%), α-guaiene (9.227%), seychellene (7.566%), etc.

**Table 5:** GC–MS analysis of the essential oils of clove bud oil and patchouli oil

Number	Compounds	Retention time (min)	Relative percentage (%)	Similarity index (NIST)
Clove bud oil				
1	Eugenol	20.119	82.093	98
2	Copaene	20.378	0.110	99
3	β-Caryophyllene	21.541	8.729	99
4	α-Caryophyllene	22.361	2.142	94
5	Acetyleugenol	23.998	4.802	98
6	Caryophyllenyl alcohol	24.880	0.089	91
7	Caryophyllene oxide	25.123	1.239	91
8	Humulene epoxide I	25.400	0.088	95
9	Humulene epoxide II	25.571	0.199	95
	Total		**99.491**	
Patchouli oil				
1	β-Pinene	8.957	0.128	91
2	δ-Elemene	19.303	0.074	97
3	Copaene	20.336	0.233	96
4	β-Patchoulene	20.503	1.738	98
5	β-Elemene	20.747	1.002	98
6	Cycloseychellene	21.238	0.854	98
7	Caryophyllene	21.487	2.829	99
8	α-Guaiene	21.965	9.227	99
9	Seychellene	22.094	7.566	99
10	α-Caryophyllene	22.356	0.633	97
11	α-Patchoulene	22.431	4.483	99
12	Patchoulene	22.559	1.415	99
13	β-Selinene	23.147	0.096	99
14	cis-β-Guaiene	23.418	1.906	99
15	α-Bulnesene	23.597	10.962	99
16	7 epi-α-Selinene	23.845	0.186	96
17	( +)-Norpatchoulenol	24.641	0.755	95
18	Caryophyllene oxide	25.118	1.897	94
19	Spathulenol	25.883	0.223	91
20	Pogostol	26.313	2.630	91
21	Patchouli alcohol	26.457	34.717	96
22	Rotundone	27.081	3.000	98
23	Pogostone	27.169	5.020	97
24	Corymbolone	29.159	0.465	98
	Total		**92.039**	

## Discussion

The minimum efficacy of EOs as mosquito repellents has been examined in only a few studies but their results are difficult to compare with our own, due to different test parameters, environmental conditions (air temperature, humidity, wind speed) (Lupi et al. 2013), test systems (Deng et al. 2014), material standards (Rehman et al. 2014), and the characteristics of the EOs, such as their rate of evaporation and the speed of their percutaneous penetration (Tavares et al. 2018). Differences in biting pressure in a mosquito population can also affect repellency testing (Barnard et al. 1998). The GB2009 test has been validated for use in evaluating mosquito repellents but it is mostly used in China, while the WHO2009 is used globally. So based on the GB2009 results obtained for the seven EOs, the two most effective EOs, clove bud oil and patchouli oil, were tested with the WHO 2009, which has the additional advantage that it allows comparisons of the results with those obtained in tests of other EOs. Normally, the better way for this study was used the same species of mosquito to test the repellency with different methods considering of their bias between the subject and bioassays or other potential influencing factors during the research. However, *Ae. albopictus* and *Ae. aegypti* which were the two major vectors of dengue fever in the world, but the former was more popular in China and the latter was more common in the world, so here we used the GB2009 to test *Ae. albopictus* make the comparison with other Asian test more easier, while for the *Ae. aegypti* were selected as test object of WHO2009, which made direct comparison and evaluation of effects with the world tests. In an earlier study (Barnard et al. 1998), clove oil at 25% conferred > 90 min of protection against *Ae. aegypti*, which was less than the CPT determined in our study. Clove oil was shown to an moderate repellent against *Anopheles* mosquitoes (Asadollahi et al. 2019). Previous studies reported that clove bud oil has a lower concentration of eugenol and a higher concentration of caryophyllene than the clove oil (Bhuiyan et al. 2012). Eugenol was shown to an effective mosquito repellent, and that the behavioral responses of mosquitoes depend significantly on the ratio of the chemical compounds in the repellent (Logan et al. 2010) and on the ability of the enantiomers to attract mosquitoes (Cook et al. 2011; Borrego et al. 2021). Our results suggest that eugenol maybe the compound largely responsible for the repellent action of clove bud oil. As for the patchouli oil, a combination of sage and patchouli oils prolonged the median complete-protection time of 270 min for *Anopheles dirus* (Peyton and Harrison) (Sutthanont et al. 2022). The repellent activity of patchouli alcohol compound was found to be most effective for repellent activity and 2.000 mg/cm^2^ concentration provided 100% protection up to 280 min against *Ae. aegypti*, *Anopheles stephensi* Liston, and *Culex quinquefasciatus*(Say), respectively (Gokulakrishnan et al. 2013). Based on this research, the patchouli alcohol maybe the major reason for the patchouli oil’s mosquito repellency although the CPT when using patchouli oil as a whole was lower than the published data.

A study of seven EOs as repellents against three species of mosquitoes determined a CPT for clove bud oil at 0.210 mg/cm^2^ of 54 min, when tested against *Ae. aegypti* (Phasomkusolsil and Soonwera 2011). By contrast, in our study, 0.094 mg/cm^2^ resulted in 60 min of complete protection. Therefore, the prior inclusion of a dose–response test may allow the use of a significantly lower EO concentration in the CPT test, in addition to avoiding possible skin irritation.

Among the seven initially tested EOs, clove bud oil showed maximum efficacy even at the lowest concentration, followed by patchouli oil. Cinnamon oil had the highest CPT, even at a concentration of 0.2%, conferring 45 min of protection; only clove bud oil provided comparable protection at this concentration. When used the keywords of clove bud oil and *Ae. albopictus* searching in the Web of Science, the reports almost all published about the clove bud oil to repel *Ae. aegypti* except one report was studied about its ovicidal and larvicidal but not repellency against *Ae. albopictus* (Bhat and Kempraj 2009). While *Ae. aegypti* proved more difficult to repel than *Ae. albopictus* (Lupi et al. 2013), a good efficacy against *Ae. aegypti* was achieved with clove bud oil even at a very low concentration; therefore, clove bud oil was more effective against *Ae. aegypti* than *Ae. albopictus*. Clove bud oil was also better than patchouli oil at its ED_50_ and conferred longer protection.

## Conclusion

The mosquito repellent activities of seven EOs were evaluated in two in vivo bioassays. In the GB2009 test, in which testing is conducted on a small area of skin, clove bud oil conferred > 2 h of protection at a concentration ≥ 10% and was therefore the most effective of the nine tested EOs, followed by patchouli oil. The minimum effective dose and CPT of clove bud oil and patchouli oil were then assessed using the WHO2009 method, in which a larger area of skin is exposed to a dose no higher than the approved safe dose. Both EOs demonstrated efficacy. However, before they can be used as mosquito repellents, additional work on their formulations is needed to lengthen their CPTs. An alternative approach may be microencapsulation. Further studies may lead to better repellents that reduce or evenly eliminate the daily nuisance of mosquitoes and, more importantly, mosquito-transmitted diseases.

## Data Availability

The authors confirm that the data supporting the findings of this study are available within the article.
